# Changes in Angiotensin Receptor Distribution and in Aortic Morphology Are Associated with Blood Pressure Control in Aged Metabolic Syndrome Rats

**DOI:** 10.1155/2016/5830192

**Published:** 2016-05-17

**Authors:** Verónica Guarner-Lans, Elizabeth Soria-Castro, Rocío Torrico-Lavayen, Araceli Patrón-Soberano, Karla G. Carvajal-Aguilera, Vicente Castrejón-Tellez, María Esther Rubio-Ruiz

**Affiliations:** ^1^Department of Physiology, Instituto Nacional de Cardiología “Ignacio Chávez”, 14080 Mexico City, DF, Mexico; ^2^Department of Pathology, Instituto Nacional de Cardiología “Ignacio Chávez”, 14080 Mexico City, DF, Mexico; ^3^National Laboratory of Nanoscience and Nanotechnology, Molecular Biology Division, Instituto Potosino de Investigación Científica y Tecnológica (IPICYT), 78216 San Luis Potosí, SLP, Mexico; ^4^Laboratorio de Nutrición Experimental, Instituto Nacional de Pediatría, 04530 Mexico City, DF, Mexico

## Abstract

The role of the renin-angiotensin system (RAS) in blood pressure regulation in MS during aging is unknown. It participates in metabolic syndrome (MS) and aging regulating vascular tone and remodeling. RAS might participate in a compensatory mechanism decreasing blood pressure and allowing MS rats to reach 18 months of age and it might form part of therapeutical procedures to ameliorate MS. We studied histological changes and distribution of RAS receptors in aortas of MS aged rats. Electron microscopy images showed premature aging in MS since the increased fibrosis, enlarged endothelium, and invasion of this layer by muscle cells that was present in control 18-month-old aortas were also found in 6-month-old aortas from MS rats. AT1, AT2, and Mas receptors mediate the effects of Ang II and Ang 1-7, respectively. Fluorescence from AT2 decreased with age in control and MS aortas, while fluorescence of AT1 increased in aortas from MS rats at 6 months and diminished during aging. Mas expression increased in MS rats and remained unchanged in control rats. In conclusion, there is premature aging in the aortas from MS rats and the elevated expression of Mas receptor might contribute to decrease blood pressure during aging in MS.

## 1. Introduction

Metabolic syndrome (MS) is a disease that accelerates aging [1]. Aging is associated with endothelial dysfunction and structural and functional vascular changes [2]. We have previously studied changes in blood pressure during aging in a MS rat model and found an increase at 6 months of age in comparison to control groups and a further decline from then onwards until 18 months of age. Rats that survive to reach 18 months are few and could be the ones that develop compensatory mechanisms to the effects of MS, the fall of arterial pressure being one of them [3, 4]. These compensatory mechanisms might be the same that underlie the condition of healthy obese or healthy MS patients and could be the basis for the development of therapeutical approaches to complications of MS. The renin angiotensin system (RAS) might participate in these mechanisms and particularly the Ang 1-7/MAS pathway.

RAS is involved in the development of MS [5] and in aging [6–8]. Moreover, long-term treatment with an angiotensin converting enzyme (ACE) inhibitor or an Ang II receptor antagonist ameliorates the MS effects [9–11]. It has long been recognized as an important regulator of systemic blood pressure and renal electrolyte homeostasis, and local RAS has also been implicated in pathological changes of organ structure and function by modulation of gene expression, growth, fibrosis, and inflammatory responses. RAS dysregulation influences the development and progression of cardiovascular disease [12].

Angiotensin II (Ang II) is a potent proinflammatory, prooxidant, and prothrombotic agent that favors endothelial dysfunction by increasing catecholamines, endothelin-1 (ET-1), and thromboxane A_2 _which increase blood pressure [13]. Ang II also alters the structure of vessels modifying processes such as growth, apoptosis, and cellular migration of vascular smooth muscle cells as well as changes in the components of the extracellular matrix [14]. It causes an increase in the intima-media thickness, vascular rigidity, endothelial dysfunction, and proinflammatory state [2]. Ang II binds to highly specific membrane receptors types 1 and 2 (AT1 and AT2). Increased levels of Ang II have been observed in both obese and diabetic patients.

Angiotensin 1–7 (Ang 1-7) is a heptapeptide, primarily formed from Ang II and Ang I by angiotensin converting enzyme 2 (ACE2) that has actions that oppose those of Ang II, acting through Mas. The components of RAS in white adipose tissue (WAT) are present in our rat model of MS by chronic ingestion of sucrose and WAT is an important source of Ang 1-7 [4]. Mas receptor is also expressed in the vascular endothelium which, at the same time, is an important site for Ang 1-7 generation [15]. The ACE2/Ang 1-7/Mas axis induces the release of vasodilators, including prostanoids, nitric oxide (NO), and endothelium-derived hyperpolarizing factor [16]. The vasodilator actions of Ang 1-7 depend on an intracellular signaling mechanism, as yet not fully characterized, that may be caused by secretion of prostacyclin, release of NO, amplification of the vasodilator effects of bradykinin, or vasopressin liberation alone or in combination [17].

Activation of endogenous ACE2 reduces arterial blood pressure in normal and hypertensive rats and relaxes many vascular beds [18]. Angiotensin 1-7 and its receptor Mas (Ang 1-7/Mas) were described later and antagonize the effect of AT2; therefore, they could represent a target for therapeutical strategies. The elevated expression of Mas receptor might contribute to decreases of blood pressure.

There is little information on the location AT1 and AT2 receptors in the different cell types of the vasculature and thus the identification of their function is complex. Moreover the belief that AT2 receptors are more significant during development than in adult life and so might regulate growth and development of blood vessels rather than having acute actions on the vasculature has delayed the study of their function in adult vessels [19]. During aging, Ang II, ACE, and AT1 increase in the vascular wall [20].

Therefore, the aim of the present study was to document the premature aging of the vessels in MS by comparing the structural changes in aortas from control and MS animals and to analyze the expression of AT1, AT2, and Mas receptors in these vessels as important components of RAS which might participate in the compensatory mechanisms that allow the 18-month-old MS rats to survive.

## 2. Materials and Methods

### 2.1. Animals

The experimental protocols in this study were reviewed and approved by the Laboratory Animal Care Committee of our institution and performed in accordance with the institutional guidelines for research and animal use.

Weanling male Wistar rats aged 25 days and weighing 50 ± 4 g were divided randomly into two groups: (1) control rats were given tap water for drinking and (2) MS rats received 30% sucrose in drinking water. Control and MS animals aged 6, 12, and 18 months were used. At least 6 animals were used per group. Animals were fed Purina 5001 rat chow (Richmond, IN)* ad libitum* under controlled temperature and a 12 : 12 h light/dark cycle.

Systolic arterial blood pressure was measured in conscious animals using the tail cuff method previously described [3].

### 2.2. Blood Samples

After overnight fasting (12 h), the animals were killed by decapitation and blood was collected. It was spun and the serum was separated by centrifugation at 600 g during 15 min at room temperature and stored at –70°C until assayed. Plasma triglycerides were measured using a commercially available kit (Randox, Laboratories LTD, Antrim, United Kingdom). Serum insulin was determined using commercial radioimmunoassay (RIA) kit specific for rat (Linco Research, Inc.; EMD Millipore, Missouri, USA). Glucose concentration was assayed using an enzymatic kit SERA-PAK^R^ Plus from Bayer Corporation (Bayer Corporation, Sées, France). Seric concentrations of Ang II and Ang 1-7 were evaluated by capillary zone electrophoresis as previously described [4]. Abdominal fat was dissected and weighted.

### 2.3. Histologic Staining, Immunohistochemistry of Angiotensin Receptors, and Image Analysis

The fixed aortic tissues were embedded in paraffin and cut into 5 *μ*m thick sections. The sections were then stained with Masson trichrome stain to reveal histological changes. For electron microscopy, the rings of aortas were obtained and immediately fixed and embedded in Epon (Epon 812 EMS); ultrathin sections were then viewed using a Jeol 10-11.

For immunohistochemistry studies, rat aorta samples were quickly frozen in Tissue-Tek (Sakura Finetek USA, Inc., Torrance, CA). Sections were fixed with acetone and were blocked with PBS/Azide 0.02%/BSA 1% for 30 min. Subsequently, sections were incubated during 2 h at room temperature with a rabbit polyclonal antibody against AT1, AT2 (1 : 50; Santa Cruz Biotechnology, Inc., Santa Cruz, CA) and goat polyclonal antibody against Mas (1 : 50; Santa Cruz Biotechnology, Inc., Santa Cruz, CA). Antibodies were detected by using goat anti-rabbit FITC (Jackson ImmunoResearch Laboratories Inc., West Grove, PA) and rabbit anti-goat Tex Red (1 : 50; Santa Cruz Biotechnology, Inc., Santa Cruz, CA) at room temperature for 45 min. The slides were mounted with a drop of mounting medium (Dako Fluorescent mounting medium). Negative controls were prepared by substituting the primary antibody with an irrelevant antibody. Images from six different sections were collected and analyzed using confocal microscopy. The samples were scanned on a confocal laser scanning microscope FV1000 (Olympus, Corporation, Tokyo, Japan) attached/interfaced to an Olympus IX81 inverted light microscope with a 20x N.A. 0.85 air objective, all times with digital zoom of 3. FITC was excited employing the 488 nm line of an Argon laser and the emitted fluorescence was detected with a 500–530 nm spectral setting. Tex Red was excited employing the 543 nm line of an Argon laser and the emitted fluorescence was detected with a 500–633 nm spectral setting. All images were obtained with the same laser power percentage and confocal aperture. The comparison of fluorescence in rat aortas between groups was done using fixed setting on the confocal microscope; confocal images were viewed, processed, and converted to TIFF format with the FV10-ASW software (Olympus Corporation, Tokyo, Japan). We used Image J software that assigned a value for green or red fluorescence intensity to every pixel and the average pixel intensity was calculated [21]. To express the intensity of fluorescence, arbitrary units were used: relative fluorescence (Mean Fluorescence Intensity (FI)) was defined as the sum of the fluorescence intensity grayscale of all pixels divided by the addition of all the image pixels. The percentage of fluorescence between comparable groups was calculated:

(1) $$\begin{array}{c} \%\text{ Relative Fluorescence}=\sum FI*\frac{100}{\sum \text{Image pixels}}. \end{array}$$

### 2.4. Statistical Analysis

Results are expressed as mean ± standard errors of the mean (SEM). When applicable (comparisons between two values; MS and controls at the same age), statistical analysis was done by Student's *t*-test. Comparisons between groups at different ages were done by analysis of variance (ANOVA) or ANOVA on ranks followed by Student-Newman-Keuls or Dunn's tests. The accepted level of statistical significance was *p* < 0.05. Data analysis was carried out using the Sigma Stat program (Jandel Scientific).

## 3. Results

Table 1 shows the differences among the groups. At six months, experimental animals developed MS characterized by increased abdominal fat, hypertension, hypertriglyceridemia, hyperinsulinemia, and insulin resistance (HOMA-IR). Aging* per se* increased intra-abdominal fat and triglycerides concentrations in control rats. In 18-month-old MS rats, weight, visceral fat, and triglycerides were higher than in young MS rats. Fasting serum glucose levels were not significantly different among the groups, but there was an increase at 18 months in MS rats. In MS rats insulin level was only significantly increased at 6 months when compared to controls; then it showed a decrease during aging.

In control rats, the arterial blood pressure was not modified during aging. At 6 months of age systolic arterial pressure was significantly elevated in MS rats when compared to control rats (Table 1). MS rats showed a continuous blood pressure decrease with age.

Table 2 shows that seric Ang II concentrations were not different in C and MS rats at 6 months of age. Therefore, this peptide does not seem to participate in the development of hypertension in the MS model. In the C group, Ang II concentration remained unchanged while in MS animals it decreased significantly during aging. Ang 1-7 concentrations showed the inverse relationship, increasing significantly in MS rats at 18 months of age. In the control group, these values were unchanged. These results coincide with those previously reported by our group [4].

### 3.1. Morphological Study of Aging in Aortas from Control and MS Rats

**Figure 1:**
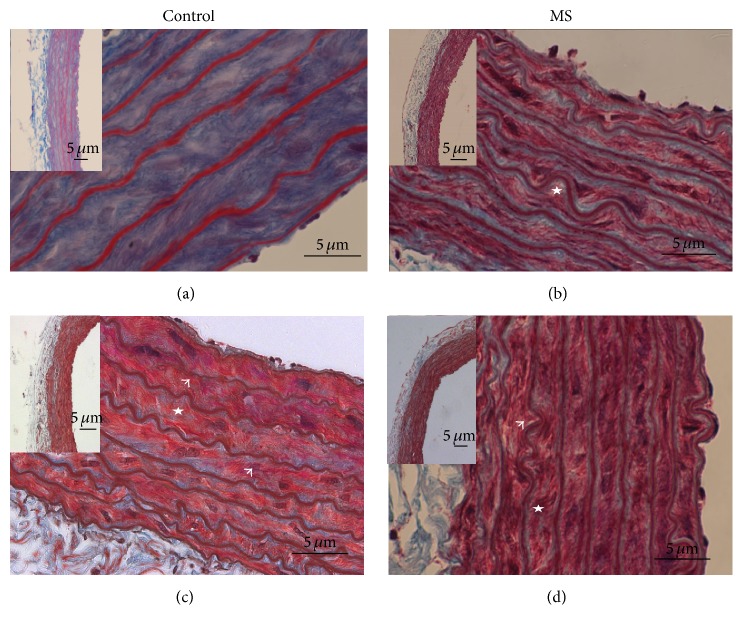
Histological images of representative rat aortas from different ages. Control ((a) 6 months and (c) 18 months) and MS ((b) 6 months and (d) 18 months) using Masson trichrome staining. The panoramic view is shown in the insert. The arrows indicate discontinuity of elastic fibers and the stars indicate hyperplasia of muscular cells.

When Masson staining was used, aortas from 18-month-old control rats showed characteristic changes due to aging when compared to 6-month-old aortas. These changes were apparent in the intima and media layers; endothelium showed swelling and in the media there was discontinuity of elastic fibers. When comparing 6-month-old aortas from control and MS rats, discontinuity of elastic fibers in the media was clear even in zones close to the intima and there was hyperplasia of muscular cells in the media in aortas from MS rats at 6 months of age. Deterioration continued even further in 18-month-old MS aortas (Figure 1). Electron microscopy images showed increased fibrosis, enlarged endothelium, and invasion of this layer by muscle cells in 18-month-old control aortas and in 6-month-old aortas from MS rats when compared to 6-month-old control aortas. In 18-month-old aortas from MS rats the endothelium was enlarged and infiltrated with cells from the immune system (Figure 1).

**Figure 2:**
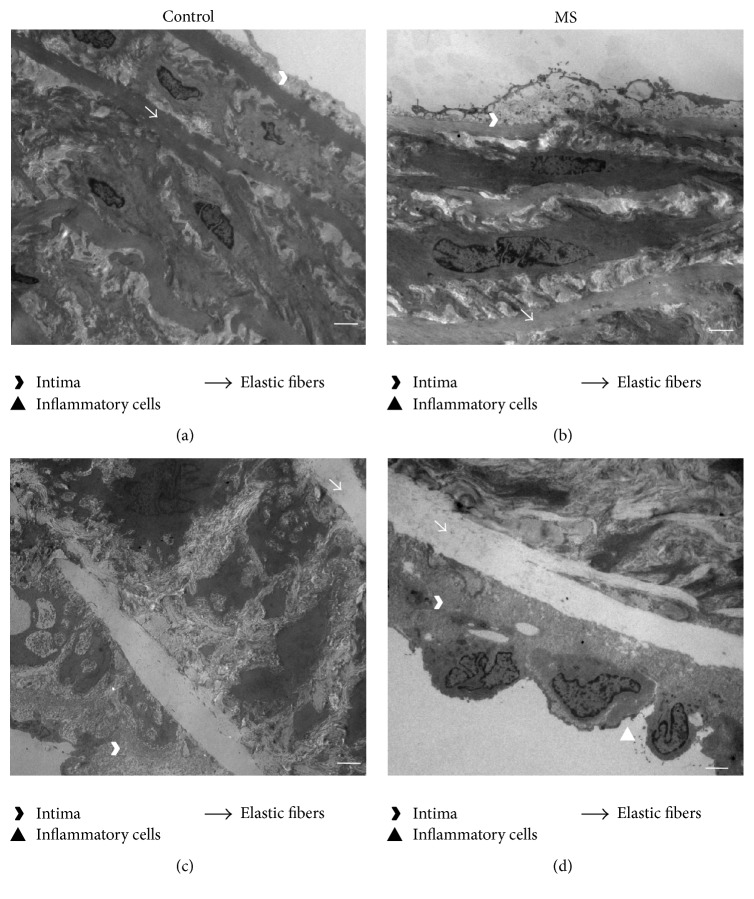
Micrographs from control 6- and 18-month-old aortas ((a) and (c), resp.) and MS 6- and 18-month-old aortas ((b) and (d), resp.). Scale bar = 2 *μ*m.

Ultrastructure studies showed differences between control and MS aortas. Aortas from 6-month-old MS rats had endothelial damage and changes in the structure of elastic fibers when compared to aortas from control animals (Figures 2(a) and 2(b)). The aging process of control aortas is seen at 18 months in which intima swelling was accompanied by cellular infiltrate which is present together with thickening of the media layer with infiltration of muscular cells. Furthermore, there is discontinuity of elastic fibers (Figure 2(c)). In the 18-month-old aortas from MS animals the above mentioned characteristics of old control vessels were also found showing adhesion of inflammatory cells in the intima layer (Figure 2(d)).

### 3.2. Angiotensin Receptor Expression in Thoracic Aorta

**Figure 3:**
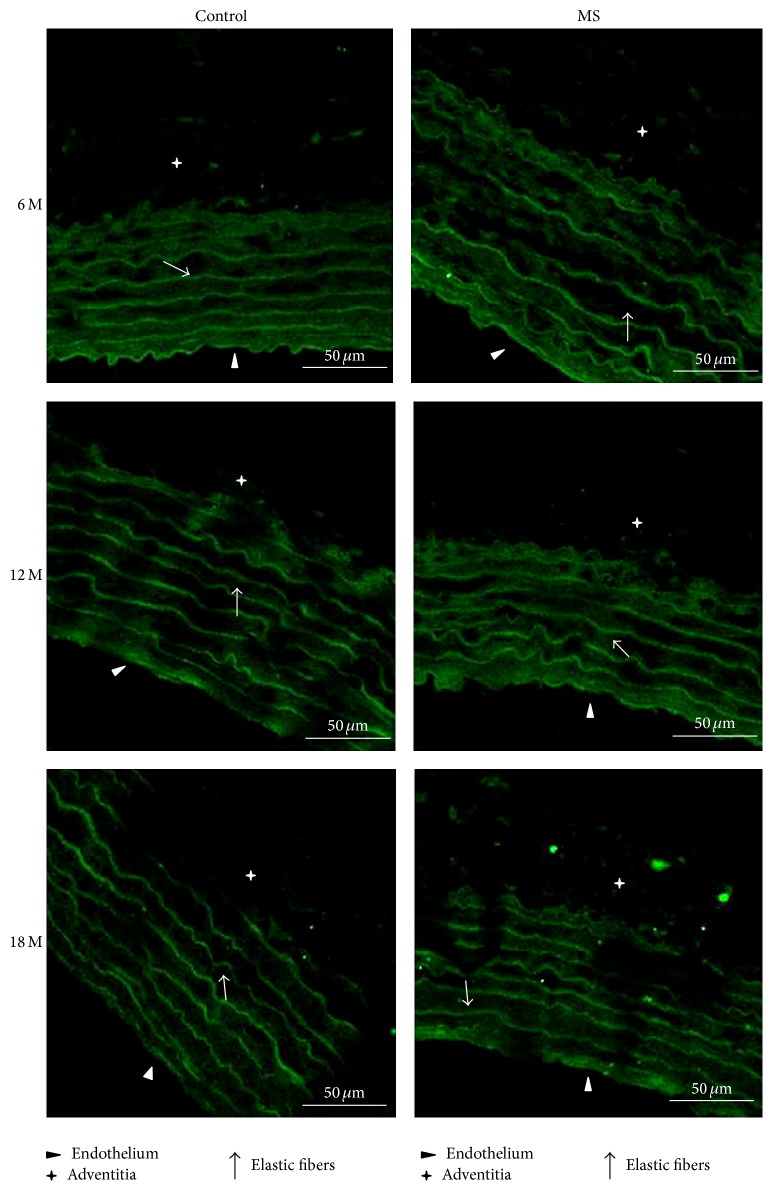
Illustrative images (×60) showing receptor AT1 immunofluorescence in aortas of control and MS rats aged 6, 12, and 18 months. Preparations were stained with FITC. Scale bar = 50 *μ*m.

The distribution of the fluorescence signal is different for each of the receptors studied. Fluorescence from AT1 receptors from aortas from 6-month-old rats was higher in the MS group than in controls. AT1 fluorescence did not change in control rats during aging and was located in the endothelium and adventitia. In contrast, in the arteries from the rats with MS, fluorescence diminishes significantly with age and it moved from the endothelium to the elastic fibers (Figures 3 and 6).

**Figure 4:**
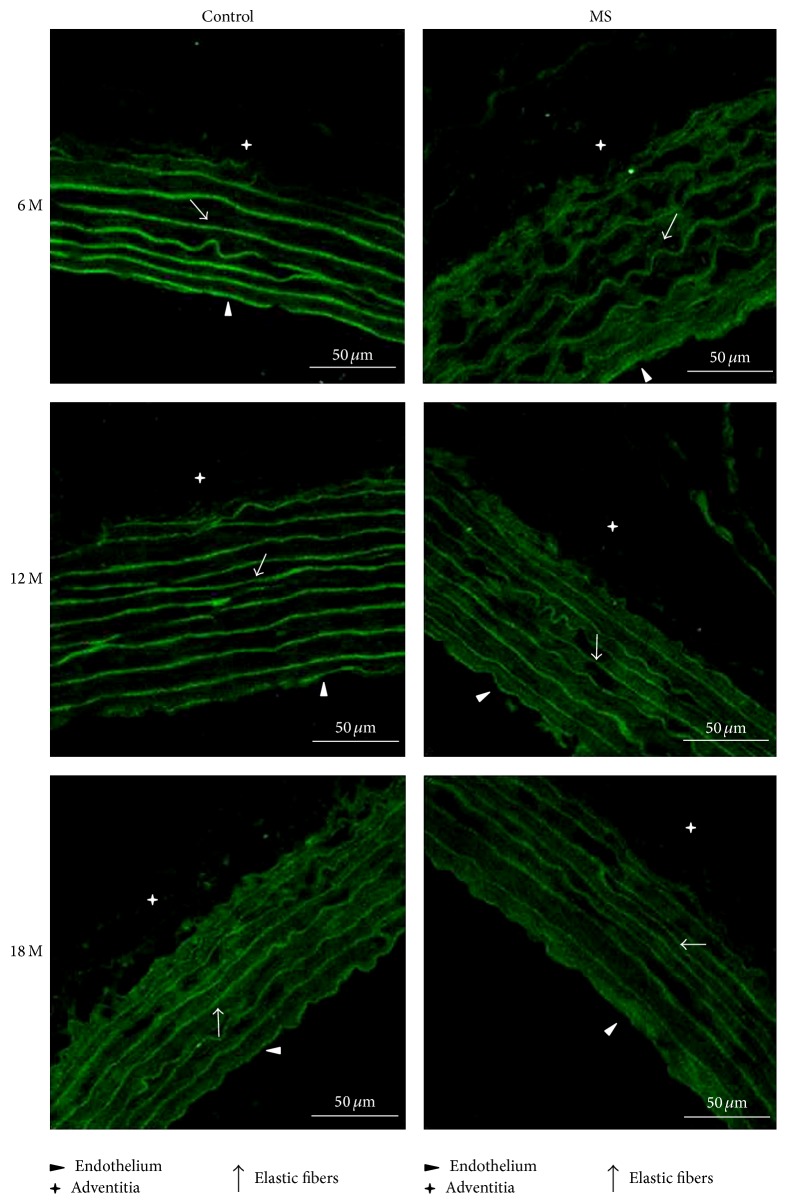
Illustrative images (×60) showing receptor AT2 immunofluorescence in aortas of control and MS rats aged 6, 12, and 18 months. Preparations were stained with FITC. Scale bar = 50 *μ*m.

Fluorescence from AT2 receptors is higher in aortas from control 6-month-old rats than in aortas from the MS group and is specifically located in the elastic fibers from the media. In old control rats, intensity decreases and is located in elastic fibers and in the endothelium. Fluorescence continues to decrease and by 18 months of age it is located in the media and adventitia. In aortas from MS rats, fluorescence is less than in control rats at all ages (especially at 18 months) and decreases with aging. At 6 months of age the signal looks like spots and is located in the elastic fibers. At 12 months, the signal is even lower and located in elastic fibers and in the adventitia. By 18 months, the fluorescence looks like spots and is only located in the endothelium and the elastic fibers (Figures 4 and 6).

**Figure 5:**
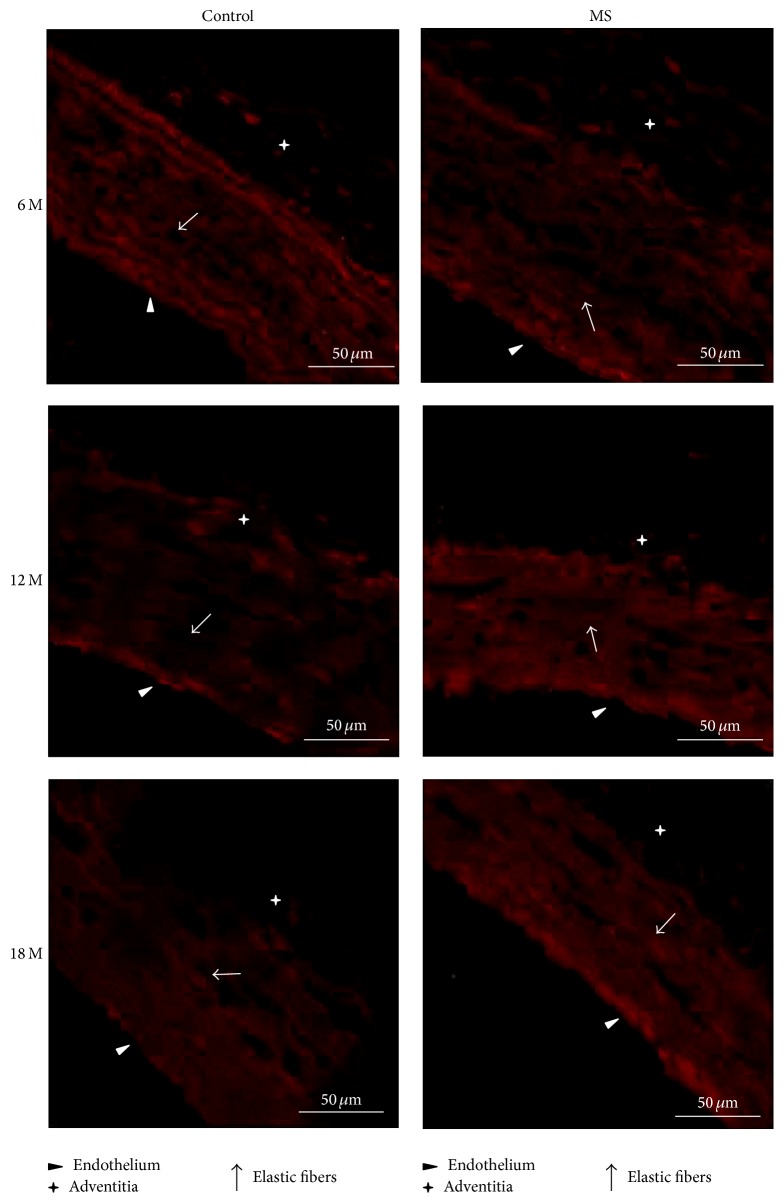
Illustrative images (×60) showing receptor Mas immunofluorescence in aortas of control and MS rats aged 6, 12, and 18 months. Preparations were stained with Tex Red. Scale bar = 50 *μ*m.

**Figure 6:**
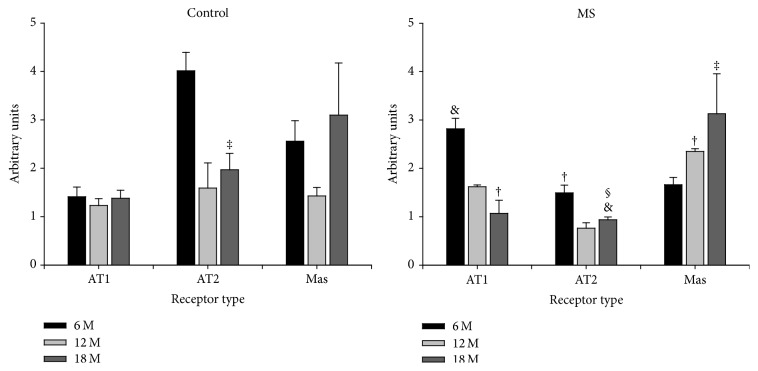
Quantitative analysis (receptor type fluorescence versus relative fluorescence) for AT1, AT2, and Mas receptors in aortas of control and MS rats during aging measured from the immunohistochemistry images in Figures 3, 4, and 5. Details of the technique are described in Section 2. Values represent mean ± SEM of six different experiments. ^&^ *P* < 0.05 against C; ^†^ *P* < 0.01 against C; ^‡^ *P* < 0.05 against other ages; ^§^ *P* < 0.05 against 6 months.

At 6 months of age, there is no difference in the fluorescence from the Mas receptors in control and MS aortas. During aging fluorescence intensity of the Mas receptor in control aortas does not change; however its distribution is modified. The signal is higher in the adventitia at 6 months and it diminishes by 12 months; it is located in the endothelium and adventitia. At 18 months the signal increases and is located in elastic fibers. In aortas from 6-month-old rats with MS, fluorescence is low, appears in spots, and is found in the endothelium and adventitia. At 12 months, fluorescence increases and is found in the adventitia and endothelium and finally by 18 months the signal is even higher and only located in the endothelium (Figures 5 and 6).

## 4. Discussion

Blood pressure is controlled by baroreflex activity that modulates sympathetic neural mechanisms and by central nervous set points that adjust its long-term level. There exist nonpathological long-term regulators that act on the central nervous set points such as aging and pathological modifiers such as obesity and heart failure. The central nervous set points receive input from volume regulatory hormones including RAS [22]. RAS constitutes a hormonal system that regulates blood pressure and its dysregulation influences the development and progression of cardiovascular disease. Although the regulation of the central nervous setpoints in our rat model might be interesting, and aging and obesity might influence their activity, in this paper we only studied the possible participation of the RAS vessel receptors during aging in control and MS rats. Ang II acts as a potent vasoconstrictor to activate AT1 receptors on vascular smooth muscle and affects cardiac and vascular remodeling, cardiac contractility, and pulse rate through increased sympathetic nervous system tone [12]. Ang 1-7 counteracts Ang II pressor and metabolic effects [16].

Our results show that rats receiving 30% sucrose in drinking water developed MS characterized by central obesity, hypertriglyceridemia, hyperinsulinemia, and moderate hypertension that are similar to those previously reported [3, 4]. Glucose was increased in MS rats at 18 months and is therefore a consequence of the aging process suggesting a prediabetic state. Aging increases the tendency to develop hyperglycemia and diabetes [23, 24].

Eighteen-month-old rats are regarded as old rats by many authors and even as senescent rats [2, 25]. Although control Wistar rats can live longer than 18 months, our model of MS rats has a significantly decreased survival rate (approximately 40%) by that age and organisms show a highly deteriorated state of health. Therefore, these rats show premature aging due to the MS. Furthermore, only rats that develop compensatory mechanisms including the capacity to restore hypertension might survive.

Blood pressure in MS rats is higher than in control animals at 6 months of age and then decreases; this might be a selection of those rats with the capacity to compensate for the hypertensive state showing chronic compensatory changes in signaling molecules such as the increase in seric Ang 1-7 concentration that may restore blood pressure and a decrease in the concentration of Ang II (Table 2). This study confirms that, in control rats, the blood pressure remains constant during aging (Table 1).

Aging is associated with impaired blood vessel function, which is an early and important event leading to cardiovascular disease [26]. Aspects of vascular aging include increased arterial stiffness, dilation of central elastic arteries, and endothelial dysfunction [27]. Dysfunction is caused by the endothelium, smooth muscle, and extracellular matrix [28] and the causes of vascular dysfunction in the endothelium and in muscle have been previously described [29–36].

The aged aortic wall exhibits a proinflammatory profile that renders it a fertile substrate for the development of arterial disease. Studies in animal models demonstrate that Ang II and its downstream signaling molecules, that is, matrix metalloproteinases and monocyte chemoattractant protein-1, increase within the diffusely thickened intima of central arteries [37].

MS is a disease that accelerates aging [38], and RAS is involved in the aging process [6–8]. An increase in the intima-media thickness, vascular rigidity, endothelial dysfunction, and a proinflammatory state has been observed in MS during aging [2]. The described aging changes shown by hematoxyline-eosin staining and electron microscopy in aortas from MS agree with previous observations in pigs [39]. Premature aging in the aortas from MS rats might be the result of changes in their aortic receptors. Structural changes, inflammation, and damage have been found in vessels during aging [40].

RAS participates both in the development of MS and in aging thus rendering the evaluation of the variations in RAS receptors important in the MS aortas. During aging Ang II, ACE, and AT1 increase in the vascular wall [41]. The vascular wall is the effector organ for hormonal or plasma RAS and AT1, AT2, and Mas receptors have been identified in the vasculature. Aortas from 6-month-old MS rats showed a higher concentration of AT1 receptor than control aortas. In control rats AT1 expression did not significantly change in the vascular wall during aging. MS increases AT1 and this receptor is low during aging. Even if there is an increased expression of AT1 in 6-month-old animals, these receptors might be inactive because the cytoplasmic domain of AT1 binds to a variety of intracellular proteins involved in receptor signaling, desensitization, and endocytosis. Furthermore, alterations in homo- or heterodimerization of AT1 with other receptors may also contribute to the pathophysiological roles [42].

At 6 months of age, the aortas of the MS animals express less amount of AT2 receptors than aortas from control animals. This decrease in the expression of AT2 could be related to the premature aging of MS animals that have a reduced vasodilator response. The distribution of the AT2 receptor in the vascular wall is nevertheless a matter of debate [43]. Advancing age is associated with arterial remodeling in diverse species. The effects of Ang II on vascular remodeling may be mediated at least in part via AT2. In this regard, AT2 may contribute to vascular adaptation and remodeling by influencing apoptosis in vascular smooth muscle cells. However, the effect of AT2 stimulation on vascular smooth muscle cells may vary between different cell phenotypes [43].

Our results show a reduction of fluorescence from AT2 in aortas from control and MS rats during aging which is consistent with the diminution found in the cardiovascular system and can be modulated in pathophysiological states associated with tissue inflammation and remodeling [44]. During aging, the fluorescence from AT2 receptor in control aortas decreases. However, we did not observe an increase in blood pressure in these animals. It is difficult to elicit a vasodilatory response directly in our aged rats; this effect may be related to AT2 receptor stimulation by Ang II. To confirm this point, we would need a more detailed description of the molecular specificity of the Ang II-AT2 pathway and to explore the possible alterations in signaling pathways in our animal model. Moreover, it has been reported that a complex interaction between Mas, AT1, and AT2 mediates the Ang 1-7 effects in blood vessels [45, 46]. The expression of Mas, which was similar in MS and controls at 6 months, increases with age only in the MS group.

Additionally, the analysis of angiotensin receptor expression by immunoblotting was done and there were no changes in AT1 and AT2 receptors in the experimental groups. Mas expression was increased in the aged MS rats (data not shown). Western blot analysis does not show the differences in the presence of the receptors in the different layers of the aortic tissue and adds little information on their location.

In conclusion, aging modifies body weight, abdominal fat, and triglycerides but these variables are further increased by MS. There is premature aging in this group since MS increases the progressive damage normally caused by aging. Alterations in the structure and function of the endothelium might be responsible of cardiovascular dysfunctions associated with aging in MS. The increase in Mas receptors coupled with the increased concentration of Ang 1-7 might explain, in part, the lowering of blood pressure in the aged MS rats. The Ang 1-7/Mas axis might be an effective therapeutic target, since the surviving rats might have developed compensatory mechanisms similar to those found in healthy obese or MS patients.

## Figures and Tables

**Table 1:** Characteristics and biochemical parameters from control and MS rats during aging.

Groups and age (months)	Body weight (g)	Central adiposity (g)	Blood pressure (mm Hg)	Glucose (mM)	Triglycerides (mg/dL)	Insulin (*μ*U/mL)	HOMA-IR
*Control*							
6	517.1 ± 6.7	6.2 ± 0.9	101.6 ± 0.9	5.0 ± 0.9	50.9 ± 5.2	5.9 ± 1.3	1.9 ± 0.7
12	580.2 ± 9.6	10.3 ± 1.4	100.0 ± 1.8	5.1 ± 0.4	96.1 ± 4.3^§^	6.1 ± 2.4	2.0 ± 0.9
18	620.9 ± 10.4^‡^	14.4 ± 3.1^§^	106.1 ± 1.1	5.4 ± 0.8	149.7 ± 6.9^‡^	6.4 ± 0.9	1.7 ± 0.9
*MS*							
6	535.7 ± 12.3	14.7 ± 0.8^†^	148.6 ± 1.2^†^	4.9 ± 0.9	113.3 ± 10.4^†^	22.4 ± 5.2^†^	4.7 ± 2.1^†^
12	650.7 ± 17.4	19.9 ± 2.1^†^	115.2 ± 5.6	5.9 ± 0.1	198.6 ± 25.7^†§^	19.6 ± 8.1	4.5 ± 1.7^&^
18	806.3 ± 18.7^†‡^	43.3 ± 5.2^†‡^	100.1 ± 2.1^‡^	6.1 ± 0.4	271.3 ± 31.4^†§^	6.9 ± 2.3^§^	1.9 ± 0.6^§^

Values are means ± SEM.; HOMA-IR: homeostatic model assessment of insulin resistance; *n* = 6; ^&^ *P* < 0.05 against C; ^†^ *P* < 0.01 versus C; ^‡^ *P* < 0.05 against other ages in corresponding group; ^§^ *P* < 0.05 against 6 months in corresponding group.

**Table 2:** Seric angiotensin II and angiotensin 1-7 concentrations from control and MS rats during aging.

Groups and age (months)	Ang II (pmol/mL)	Ang 1-7 (pmol/mL)
*Control*		
6	0.09 ± 0.02	2.31 ± 0.17
12	0.05 ± 0.01	4.21 ± 0.41
18	0.06 ± 0.01	3.16 ± 1.29
*MS*		
6	0.11 ± 0.03	2.61 ± 0.36
12	0.09 ± 0.01^&^	3.97 ± 0.41
18	0.008 ± 0.0006^&‡^	7.91 ± 0.46^†‡^

Values are means ± SEM; *n* = 6; ^&^ *P* < 0.05 against C;^  †^ *P* < 0.01 versus C; ^‡^ *P* < 0.05 against other ages in corresponding group.
